# Comprehensive Diagnostic Assessment of Inverter Failures in a Utility-Scale Solar Power Plant: A Case Study Based on Field and Laboratory Validation

**DOI:** 10.3390/s25123717

**Published:** 2025-06-13

**Authors:** Karl Kull, Bilal Asad, Muhammad Usman Naseer, Ants Kallaste, Toomas Vaimann

**Affiliations:** 1 Evecon OÜ, Lossi Street 3, 93819 Kuressaare City, Estonia; karl.kull@evecon.ee; 2 Department of Electrical Power Engineering and Mechatronics, Tallinn University of Technology, 19086 Tallinn, Estonia; muhammad.naseer@taltech.ee (M.U.N.); ants.kallaste@taltech.ee (A.K.); toomas.vaimann@taltech.ee (T.V.)

**Keywords:** arc faults, fault diagnosis, field monitoring, grid-connected systems, photovoltaic systems, power inverters, power system protection, power quality, power reliability, short-circuits, semiconductor device failure

## Abstract

Recurrent catastrophic inverter failures significantly undermine the reliability and economic viability of utility-scale photovoltaic (PV) power plants. This paper presents a comprehensive investigation of severe inverter destruction incidents at the Kopli Solar Power Plant, Estonia, by integrating controlled laboratory simulations with extensive field monitoring. Initially, detailed laboratory experiments were conducted to replicate critical DC-side short-circuit scenarios, particularly focusing on negative DC input terminal faults. The results consistently showed these faults rapidly escalating into multi-phase short-circuits and sustained ground-fault arcs due to inadequate internal protection mechanisms, semiconductor breakdown, and delayed relay response. Subsequently, extensive field-based waveform analyses of multiple inverter failure events captured identical fault signatures, thereby conclusively validating laboratory-identified failure mechanisms. Critical vulnerabilities were explicitly identified, including insufficient isolation relay responsiveness, inadequate semiconductor transient ratings, and ineffective internal insulation leading to prolonged arc conditions. Based on the validated findings, the paper proposes targeted inverter design enhancements—particularly advanced DC-side protective schemes, rapid fault-isolation mechanisms, and improved internal insulation practices. Additionally, robust operational and monitoring guidelines are recommended for industry-wide adoption to proactively mitigate future inverter failures. The presented integrated methodological framework and actionable recommendations significantly contribute toward enhancing inverter reliability standards and operational stability within grid-connected photovoltaic installations.

## 1. Introduction

**Figure 1:**
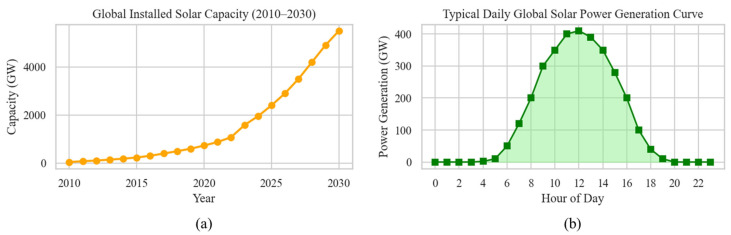
Trends and global landscape of solar photovoltaic (PV) deployment. (**a**) Global cumulative installed solar PV capacity from 2010 to 2030. Historical data from 2010 to 2023 are based on IEA and IEA-PVPS reports, while projections from 2024 to 2030 reflect current deployment trends and global energy transition targets. (**b**) A typical daytime solar power generation profile under clear-sky conditions, highlighting peak generation around midday and zero output during nighttime.

The global energy landscape is witnessing an unprecedented shift toward renewable energy sources, with solar photovoltaic (PV) systems emerging as one of the fastest-growing segments within this sector. Driven by increasing environmental concerns, supportive policies, and technological advancements, global solar PV capacity has expanded dramatically over the past decade, surpassing 1 terawatt by 2022 and continuing a trajectory of rapid growth [1], as shown in Figure 1.

Solar power plants ranging from small residential installations to large-scale utility projects now constitute a significant share of electricity generation worldwide. However, the rapid adoption of solar power systems has introduced considerable challenges, particularly concerning the reliability of critical components such as inverters [2,3]. Despite technological progress, inverter failures continue to pose substantial hurdles, frequently leading to extended downtime, reduced plant productivity, and escalated operation and maintenance (O&M) costs. Recent industry surveys indicate that inverter malfunctions account for a substantial proportion of the total PV system failures, underscoring the urgent need for deeper technical investigations into inverter reliability issues [4].

Inverters play a crucial role in PV systems, acting as the interface between direct current (DC) generated by solar panels and the alternating current (AC) required by the grid. Consequently, inverter reliability is directly linked to the operational efficiency, economic profitability, and stability of solar power plants [5,6]. Unreliable inverter operation not only impacts the financial returns due to increased downtime and higher maintenance expenditures but also poses significant threats to grid stability. Faulty inverter operations, including internal short-circuits and component failures, can induce power quality disturbances such as voltage dips, harmonic distortions, and asymmetries, potentially affecting broader network operations [7,8]. For grid operators and solar plant owners alike, the tasks of understanding and mitigating inverter reliability risks are imperative in order to maintain a continuous, high-quality power supply and ensure compliance with strict power quality standards, thus guaranteeing economic sustainability and the grid-compatibility of solar investments.

Inverter failures in PV power plants can result from a complex interplay of technical factors, many of which have been insufficiently explored or documented, especially in real-world conditions. Typical technical reasons encompass both internal design vulnerabilities and external operating environment anomalies [9,10]. For instance, internal faults may include failures of critical switching components, such as insulated-gate bipolar transistors (IGBTs), caused by excessive thermal stress, prolonged operation beyond rated specifications, or inadequate internal protection mechanisms. Externally induced failures often involve detrimental power quality issues such as transient voltage surges, severe harmonic disturbances, voltage asymmetries, and rapid voltage fluctuations that exceed the inverter’s designed operational thresholds [11,12,13]. Additionally, environmental factors like moisture ingress due to improper sealing or manufacturing errors can precipitate insulation breakdowns, leading to catastrophic internal arcing [14]. Such arcs may escalate rapidly into severe multi-phase short-circuits, causing irreversible thermal and structural damage within the inverter modules. Recent investigations have identified DC side grounding faults as particularly problematic; these can initiate catastrophic chain reactions resulting in widespread component destruction [15,16]. Addressing these technical challenges requires meticulous analysis and validation, highlighting the necessity of robust diagnostic approaches that combine field measurements with controlled laboratory simulations to uncover precise root causes and failure mechanisms, ultimately guiding improvements in inverter design and operational strategies.

Several case studies emphasize specific vulnerabilities of inverter DC-link circuits, highlighting scenarios in which DC-side short-circuits or grounding faults propagate rapidly into incidents of severe internal damage, compromising semiconductor components and protective circuitry [17,18,19]. Moreover, the literature indicates that improper handling of transient grid disturbances, such as with inadequate Fault Ride Through (FRT) functionality, frequently exacerbates damage severity during transient events [20,21]. Recent investigations have also emphasized environmental factors, including moisture ingress due to manufacturing defects or improper sealing, as critical initiators of insulation breakdowns, leading to catastrophic internal arc faults and subsequent inverter destruction.

Despite the considerable attention these reliability concerns have received, there remains a notable lack of integrated methodologies that combine laboratory-controlled fault reproduction with comprehensive field diagnostics. This methodological gap has constrained the precise identification of failure pathways and hindered the development of robust inverter protection solutions. Therefore, addressing this gap by integrating controlled simulations and field-validation efforts, as executed in this paper, represents a significant advancement toward enhanced understanding and mitigation of inverter reliability issues.

In response to the global challenges discussed above, this paper closely investigates a series of critical inverter failures at the Kopli Solar Power Plant located in Valga County, Estonia. Despite the use of the contemporary and widely deployed Sungrow SG125HX multi-MPPT string inverters—certified according to rigorous international standards—the Kopli plant has experienced multiple catastrophic inverter breakdowns, characterized by incidents of extensive thermal and structural damage. Initial observations indicated severe physical deterioration in critical inverter components, notably isolation relays, IGBTs, and internal DC-link capacitors. Additionally, pronounced evidence of internal arcing and soot deposition further underscored the severity of these incidents. Interestingly, such destructive failures were repeatedly observed under seemingly stable operating conditions, raising significant concerns regarding the actual resilience of inverter internal protection schemes and external grid interactions. Preliminary analyses conducted during field inspections identified the potential roles of DC-input grounding faults and inadequate internal protective mechanisms in amplifying the damage. Given the frequent recurrence and severity of these incidents, a rigorous technical exploration was deemed essential not only to ascertain precise failure mechanisms but also to inform preventative measures for similar grid-connected PV systems.

Motivated by the complexity and practical significance of these inverter failure scenarios, this paper aims to systematically dissect the mechanisms underlying recurrent catastrophic inverter failures through an innovative, dual-validation approach. The objectives of the presented research are threefold.

- First, we meticulously recreated critical inverter fault conditions within a controlled laboratory environment, utilizing comprehensive short-circuit simulations on DC input terminals to precisely replicate the conditions hypothesized to trigger the observed field failures. This controlled setting enabled direct analysis of inverter internal responses under simulated catastrophic conditions, providing unique insights into their protective behavior.
- Second, detailed field monitoring was performed at the Kopli Solar Power Plant, employing advanced power quality analyzers and capturing critical fault signatures, voltage fluctuations, transient current events, and harmonic disturbances, thus providing empirical validation for the laboratory-based hypotheses. Integrating these laboratory simulations with field observations allows us to conclusively identify specific failure pathways and associated shortcomings in inverter protection mechanisms.
- Lastly, drawing from this integrated validation approach, the paper presents comprehensive recommendations aimed at improving inverter design robustness, protection algorithms, and operational guidelines, aiming to significantly mitigate future failures.

To the best of our knowledge, this integrated methodological framework—combining rigorous laboratory experimentation with detailed field validation—has not previously been applied at this scale in the published literature, making this contribution particularly valuable for researchers, manufacturers, and PV plant operators seeking enhanced reliability and operational resilience.

## 2. Background and System Description

The utility-scale photovoltaic (PV) system under investigation (Kopli Solar Power Plant, Estonia) features a high-density solar array architecture designed to ensure optimal energy harvesting and efficient grid interfacing.

The plant occupies two different locations and incorporates a large-scale solar photovoltaic (PV) consisting of approximately 8960 PV modules arranged into multiple strings, each comprising 28 modules connected in series. Ten such strings are connected in parallel to a single inverter, yielding a total of 32 inverters tasked with power conversion across the facility.

These inverters operate under a three-phase configuration, delivering alternating current (AC) output at a rated voltage of 3 × 630/800 V (50 Hz) through standard AXPK power cables. The conversion process is managed by multi-MPPT string inverters designed to handle substantial DC input voltages while maintaining high efficiency and tracking performance across multiple irradiance conditions.

To enable seamless power distribution and grid compliance, the AC outputs from the inverters are fed into centralized medium-voltage substations configured with transformer stages operating at 0.8/10.5 kV. These substations serve as key interfacing points, aggregating distributed inverter outputs and preparing them for medium-voltage transmission. A dedicated 10 kV underground cable line is used to export the generated energy to the grid interconnection point, supporting a maximum injection capacity of up to 4 MW.

**Figure 2:**
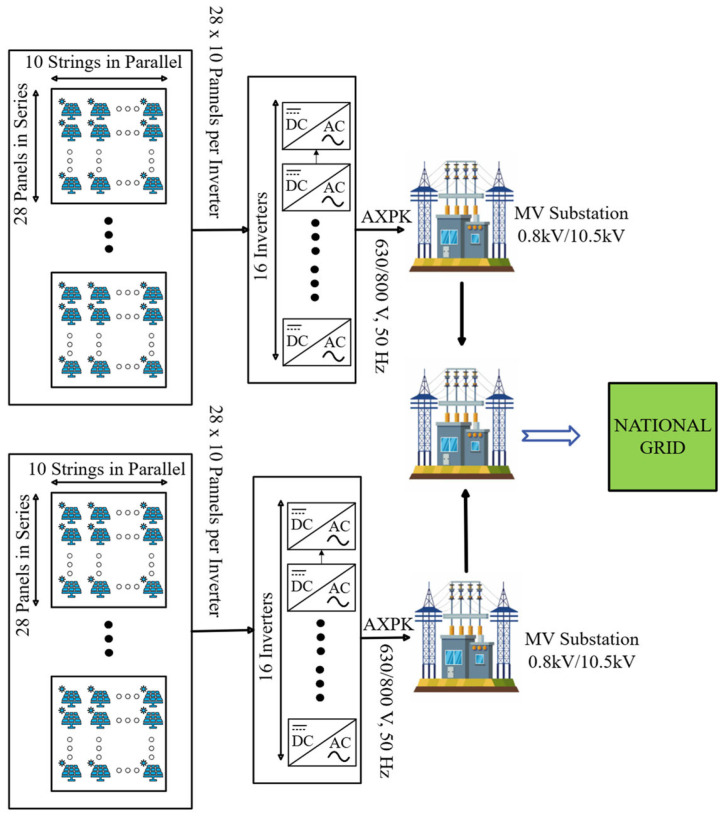
System-level architecture of the utility-scale PV plant, showing modular PV strings connected to multi-MPPT string inverters, which interface with medium-voltage substations operating at 0.8/10.5 kV. The aggregated output is transmitted via a 10 kV feeder to the grid interconnection point, enabling centralized power export and grid compliance.

The plant’s power flow configuration, modular inverter integration, and substation interconnectivity are schematically represented in Figure 2, illustrating a robust and scalable system layout suitable for high-penetration solar deployment.

**Figure 3:**
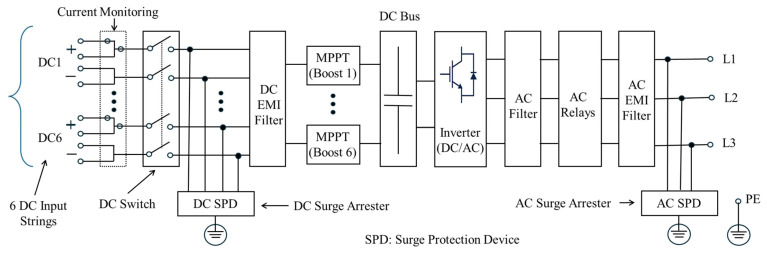
Internal layout of the SG125HX inverter, highlighting MPPT inputs, DC-link stage, switching bridge, isolation relays, and EMI filtering components.

The facility employs Sungrow SG125HX inverters, each rated at 125 kVA and manufactured by Sungrow Power Supply Co., Ltd., Hefei, China. These multi-MPPT string inverters are engineered for 1500 V DC PV applications and incorporate twelve PV string inputs, grouped into six independent MPPT channels. Internally, each inverter integrates critical components, including IGBT-based switching modules, DC-link capacitors, isolation relays, surge protection devices, and high-frequency electromagnetic interference (EMI) filters, as shown in Figure 3. On the AC side, the inverter includes additional isolation relays and low-frequency filtering stages to ensure compliance with grid interconnection standards and to minimize harmonic emissions.

Despite these comprehensive design features and its planners having observed strict design standards, the repeated, catastrophic inverter failures observed in Kopli highlight critical vulnerabilities; these have motivated the present in-depth investigation into potential internal and external failure mechanisms. The integration of grid-connected PV inverters, such as the Sungrow SG125HX utilized at the Kopli plant, mandates strict compliance with various international technical standards and regulatory norms. These standards ensure safe operation, grid compatibility, electromagnetic compliance, and robust inverter performance under diverse electrical conditions. Specifically, the SG125HX inverter has been certified according to multiple global standards, including IEC 62109 (Safety of power converters for PV systems), IEC 61727 (PV systems—Characteristics of utility interface), IEC 62116 (Islanding prevention measures), IEC 61000 series (Electromagnetic compatibility), and VDE-AR-N 4110:2018 and 4120:2018 (Technical Connection Rules for Medium- and High-Voltage Networks, respectively). Additionally, the inverter meets essential directives under the European Union CE marking, including conformity to the EU’s Low Voltage Directive (LVD, EN 62109-1:2010; EN 62109-2:2011) and Electromagnetic Compatibility Directive (EMC, EN 61000-6-2:2019; EN 61000-6-4:2019). Furthermore, specialized certifications such as VDE 0126-1-1, EN 50549-2, and relevant engineering guidelines (e.g., EREC G99) specify rigorous operational benchmarks for grid-connected PV systems operating in medium-voltage environments. Compliance with these extensive technical norms underscores the theoretical resilience of inverter operation; yet the recurrent catastrophic inverter failures at Kopli reveal the practical limitations of such compliance under complex, real-world operational scenarios.

## 3. Initial Inverter Failure Observations and Hypotheses Formulation

A detailed visual assessment was conducted on four severely damaged SG125HX inverters to investigate the nature and consistency of internal failures. The inspection revealed a recurring damage pattern across all units, indicating a systemic failure mechanism likely initiated by high-energy internal arcing. Thermal degradation of the AC-side isolation relays was consistently observed, with all units showing extensive damage across multiple phases. Melted plastic casings, charred residue, and soot accumulation around the relay compartments suggested sustained electrical arcing and relay contact welding. In several instances, the damage extended to adjacent filtering and connection interfaces, implying that the relay malfunction played a central role in failure propagation. The catastrophic breakdown of the inverter’s semiconductor switching bridge was also evident.

**Figure 4:**
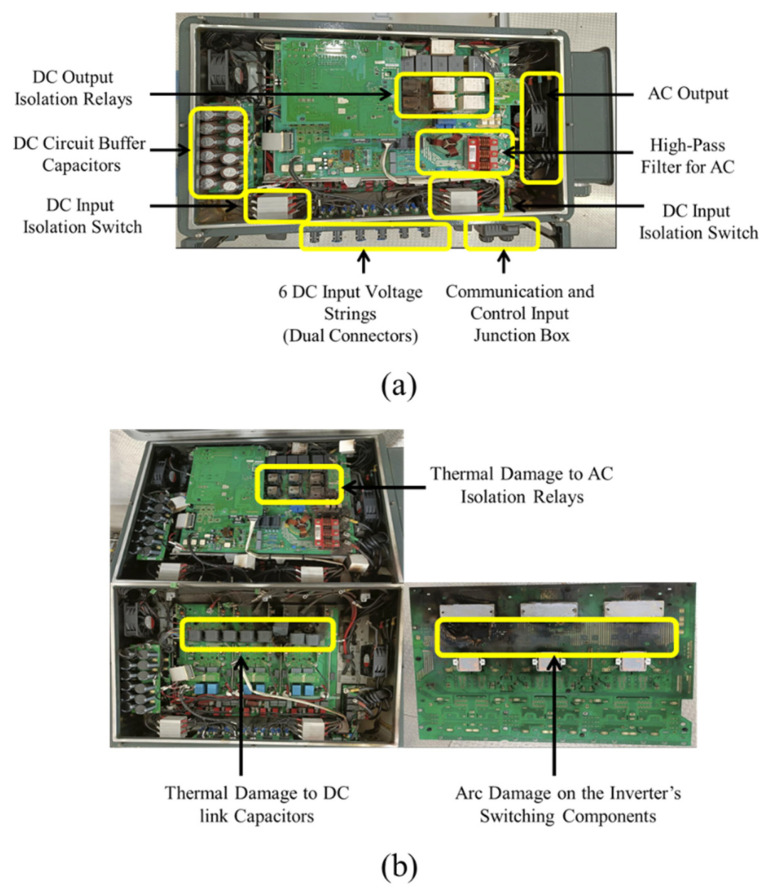
Internal configuration and faulty components of the Sungrow SG125HX inverters at the Kopli Solar Power Plant. (**a**) Annotated internal layout of a healthy inverter, highlighting key components: IGBT switching modules, DC-link capacitors, isolation relays, EMI filters, control boards, and the cooling system. (**b**) Comparative view of three failed inverter units showing recurring damage signatures, including thermal destruction of relays, ruptured DC capacitors, and arc-induced degradation of semiconductor modules and PCB tracks.

In all examined units, at least two of the three phases showed exploded IGBT packages, burnt PCB traces, and visible arc marks, indicating high-current fault paths and localized overheating. These features are consistent with sudden short-circuit events likely driven by insulation breakdown or internal conductive bridges. Further examination revealed failure of the DC-link capacitors and significant damage to the AC-side electromagnetic interference (EMI) filters. The capacitors displayed thermal expansion, ruptured casings, and electrolyte leakage, while the EMI filters exhibited extensive carbonization and heat discoloration. These signs point to high transient energy dissipation within confined zones of the enclosure, further supporting the hypothesis of rapid internal arcing as the failure trigger. Figure 4 summarizes the common failure modes observed across all four inverters, highlighting damaged relay elements, switching devices, and filtering components.

Based on the uniformity and extent of the internal damage observed across the inspected inverters, the following hypotheses were formulated to investigate potential root causes:

- Deviations in Operating Voltage and Power Quality: It was initially considered that voltage fluctuations—such as sags, swells, or harmonic distortions—could exceed the inverter’s specified tolerances and initiate fault conditions. However, long-term monitoring data indicate that voltage levels, harmonics, and phase asymmetry remained within acceptable industry and manufacturer-specified limits during the observation period, making this hypothesis unlikely.
- Protection Relay Coordination Failures: Malfunction of the AC-side isolation relays during transient disturbances may have prevented timely disconnection, resulting in sustained arcing and thermal degradation. Evidence of severe relay damage and arc signatures across all units supports the likelihood of relay mis-operation as a contributing factor.
- DC Input Ground Faults and Insulation Breakdown: Electrical breakdowns within the DC input stage—potentially due to conductor insulation failure or improper cable termination—could lead to internal short-circuits. This failure mode is supported by destructive inspection findings showing charred DC-link regions and semiconductor damage consistent with high-current DC faults.
- Moisture-Induced Dielectric Failure: Condensation or moisture ingress into the inverter enclosure, possibly due to inadequate sealing or installation deficiencies, may reduce internal insulation resistance. This could initiate localized dielectric breakdowns and arc propagation. The timing of failures—often during early daily startups—and the visual evidence of arc damage near component interfaces support this hypothesis as a credible trigger mechanism.

Each hypothesis was formulated based on the visual inspection outcomes and initial knowledge of inverter construction, operation, and grid integration dynamics. While all proposed hypotheses warranted consideration, detailed visual analysis and component-level damage characterization strongly supported certain scenarios as being more plausible. In particular,

- The hypothesis regarding DC Input Fault Conditions emerged as being particularly plausible due to the clear evidence of extensive semiconductor and DC-link capacitor damage, indicative of rapid, high-energy electrical faults originating from the DC side of the inverter circuitry. The physical ruptures and extensive arcing damage observed strongly suggested internal short-circuiting events consistent with DC-side faults propagating rapidly to inverter output stages.
- Additionally, Protection Relay Malfunctions were deemed to be highly plausible based on the incidents of severe thermal damage consistently observed in isolation relays. These incidents of damage were indicative of prolonged electrical arcs, suggesting relays either failed to open effectively under short-circuit conditions or were re-energized prematurely due to internal control logic errors.

Conversely, while environmental conditions such as moisture ingress could not initially be excluded, the extreme nature of the event and the instantaneous energy release evidenced in semiconductor destruction suggested a primarily electrical rather than purely environmental root cause. Furthermore, preliminary analysis of grid operating parameters, as discussed in the following sections, initially cast doubt on the voltage fluctuations hypothesis, shifting investigative emphasis towards internal control, relay operation, and DC-side short-circuit mechanisms. These initial insights directed the subsequent experimental and analytical investigation toward verifying and conclusively determining the exact fault mechanisms responsible for the observed inverter destructions.

## 4. Test Rig Preparation and Inverter Fault Simulations

The catastrophic inverter failures observed at the power plant under consideration necessitated an in-depth understanding of underlying fault mechanisms before comprehensive field measurements could conclusively validate their causes. Considering the substantial severity and repetitive nature of these inverter destructions, controlled laboratory experiments were first conducted. Laboratory tests provided a highly controlled and reproducible environment, enabling precise replication of suspected fault conditions, isolation of inverter-specific responses, and direct observation of internal protection mechanisms. This step was essential to clearly distinguish inverter-internal vulnerabilities from external grid-side disturbances. Hence, laboratory simulation of suspected DC input fault conditions was chosen as the foundational investigative method, providing robust preliminary data that subsequently guided targeted field-validation efforts. Laboratory simulations were meticulously planned and executed, ensuring replication of realistic operational scenarios. The experimental setup aimed to emulate inverter conditions similar to the actual grid-connected configuration at the Kopli Solar Power Plant.

**Figure 5:**
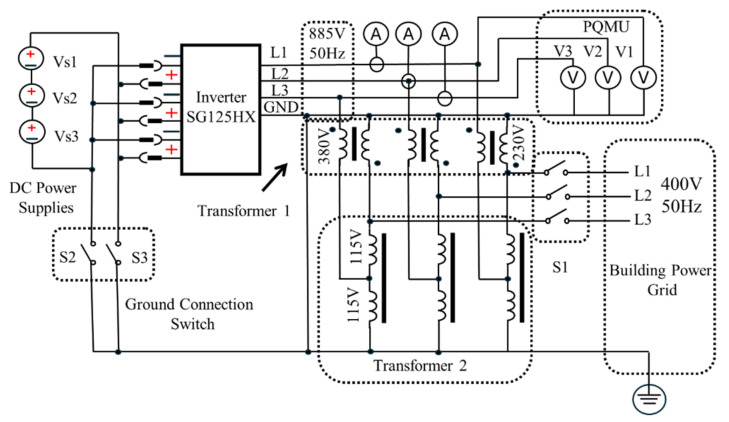
Laboratory test setup for inverter fault simulation, showing isolation transformer T1 in autotransformer configuration and transformer T2 in star–star (Yny) connection. The arrangement establishes nominal 800 V AC grid-like conditions for evaluating inverter response under controlled fault scenarios.

To replicate the realistic operating conditions for the inverter under test, a laboratory setup was constructed using two isolation transformers to achieve the required AC-side nominal voltage of 800 V ± 10%, as shown in Figure 5. Transformer T1 was configured in an autotransformer arrangement with a nominal current rating of 187 A, while transformer T2 was connected in a star–star (Yny) configuration and rated at 130 A. This combination effectively simulated grid-like voltage and phase conditions on the AC side of the inverter, ensuring stable and representative test conditions for controlled fault injection and performance evaluation.

To emulate realistic DC-side conditions, laboratory tests employed a sophisticated combination of DC power supplies. A Magna-Power TSD800-18/380 DC source was configured to replicate the characteristic PV V-I curves, thus enabling effective operation of the inverter’s built-in MPPT functionality. Three additional TDK/LAMBDA power supplies, each with a 240 V constant-voltage output, were series-connected to ensure a suitable DC voltage level capable of initiating inverter operation and sustaining an approximately 10 kW power output during testing. A high-precision power quality analyzer was utilized to comprehensively record inverter operating parameters and critical fault waveforms. Instantaneous AC voltages, currents, transient responses, and inverter operational behavior during fault events were precisely captured, ensuring robust analytical capabilities. To replicate suspected failure mechanisms, focused short-circuit tests were carried out on inverter DC inputs:

- Negative DC Input to Ground Short-Circuit Tests: Multiple repeated short-circuit tests were conducted on the negative DC input terminal, simulating scenarios suspected to trigger severe inverter failures. Each test involved the deliberate grounding of the DC negative terminal via a controlled contactor switch, instantly inducing high-energy fault conditions within the inverter circuitry.
- Positive DC Input to Ground Short-Circuit Tests: Complementary tests were performed by grounding the positive DC input terminal under identical conditions to distinguish inverter responses in terms of negative and positive terminal fault conditions, further clarifying inverter protection behavior.

**Figure 6:**
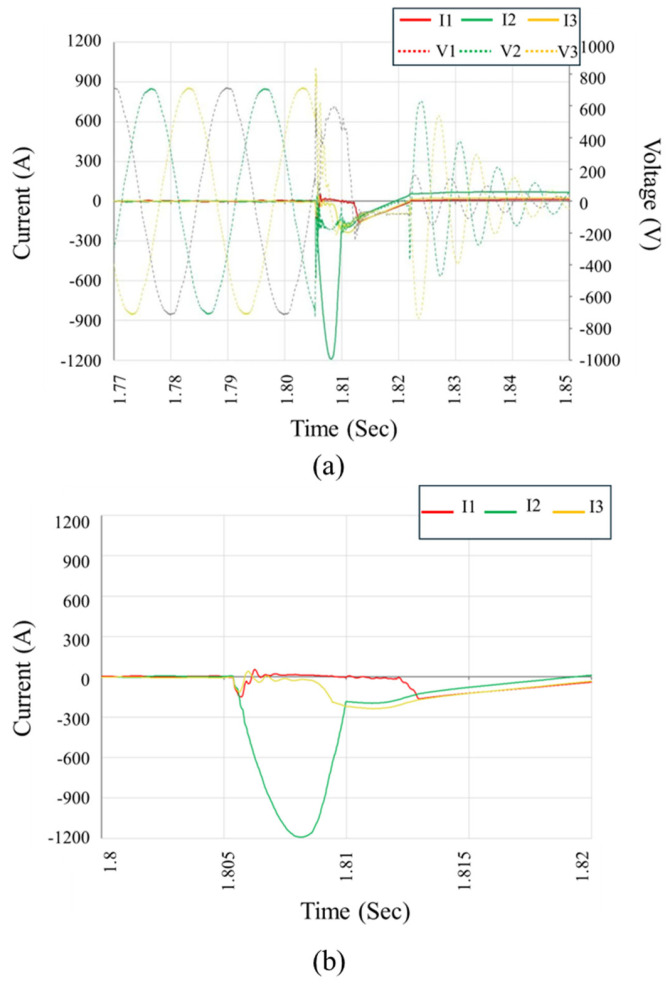
Measured voltage and current waveforms during laboratory fault simulation, illustrating inverter response under controlled short-circuit conditions. Waveforms highlight the onset of the fault, transient behavior, and protection system activation in (**a**) while zoomed version of current waveforms is shown in (**b**).

The detailed waveform analysis and inverter responses obtained from these tests provided critical insights. Short-circuiting the negative DC input consistently triggered severe fault currents in the inverter’s AC-side circuitry, with instantaneous phase current magnitudes exceeding 1 kA, as shown in Figure 6. Notably, the inverter protection systems repeatedly failed to mitigate these faults in a timely manner, resulting in immediate and sustained overcurrent conditions, followed by rapid circuit-breaker activation due to extreme stress. These findings strongly correlated with the observed semiconductor and relay damage identified in field-failed units.

Conversely, positive DC terminal short-circuits did not generate catastrophic internal currents. Instead, inverter protective mechanisms functioned appropriately, immediately disconnecting DC-side power sources without generating severe internal faults. These results clearly indicated an asymmetric inverter protection response, specifically highlighting vulnerabilities associated with negative DC terminal grounding faults. Laboratory tests conclusively identified negative DC-side short-circuits as critical events capable of triggering catastrophic inverter destruction through sustained internal overcurrent and semiconductor device breakdown. The inability of the inverter’s internal protection circuitry to mitigate these faults highlighted a fundamental design vulnerability that required immediate field verification. These controlled laboratory findings provided targeted guidance for subsequent detailed field analysis, focusing specifically on detecting and analyzing events correlated with DC-side fault signatures, thus ensuring the precise and practical validation of identified inverter failure mechanisms under real-world operating conditions.

## 5. Field Measurement and Power Quality Monitoring Methodology

**Figure 7:**
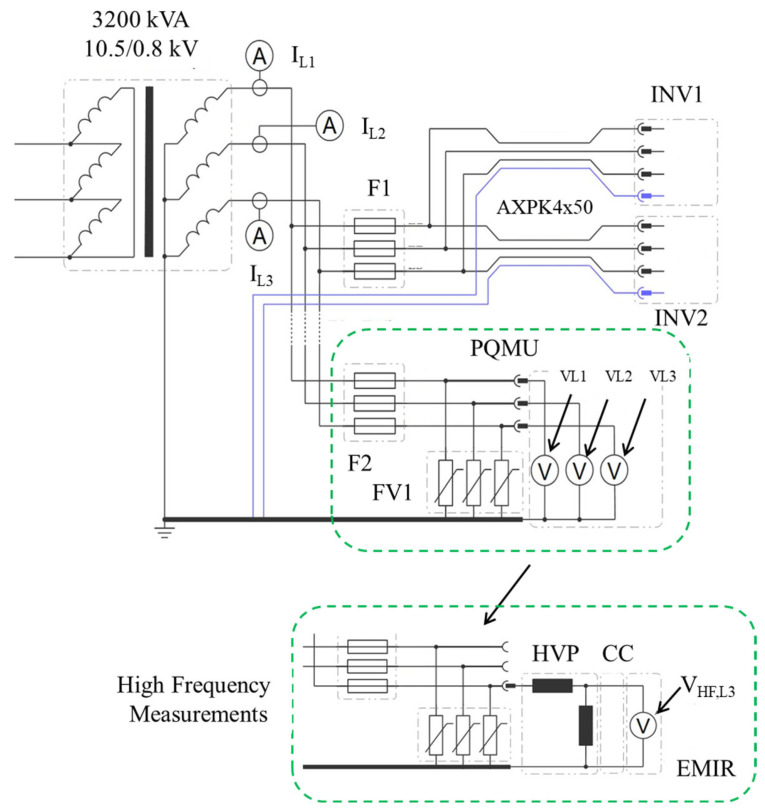
Power quality and high-frequency measurement setup at the Kopli Solar Power Plant Substation. The system includes current and voltage sensing on the 10.5/0.8 kV transformer secondary side using a power quality monitoring unit (PQMU) connected via surge arrestor feeders. High-frequency conducted emissions are measured using a high-voltage probe (HVP), current clamp (CC), and EMI receiver (EMIR), enabling detailed analysis of transient and electromagnetic disturbances from inverters INV1 and INV2.

Following conclusive laboratory simulations, extensive field measurements were conducted at the Kopli Solar Power Plant to validate identified inverter fault mechanisms under real-world operational conditions. A detailed on-site monitoring strategy was formulated to comprehensively capture critical operational variables, inverter behavior, and power-quality parameters influencing inverter reliability. A high-precision power quality analyzer (PQMU) was strategically installed at the Kopli Substation, enabling continuous monitoring and detailed data acquisition for inverter performance during normal and abnormal operating conditions, as shown in Figure 7. Voltage measurement connections were established via dedicated banana-type connectors integrated into existing surge arrester feeders, ensuring safe and accurate measurement points. Current measurements employed non-invasive current clamps connected directly to secondary circuits of existing current transformers in the substation, thus preserving original circuit integrity and safety protocols. The analyzer was configured to adhere strictly to the IEC 61000-4-30 standard, facilitating the reliable and standardized measurement reporting suitable for in-depth power quality assessments.

**Figure 8:**
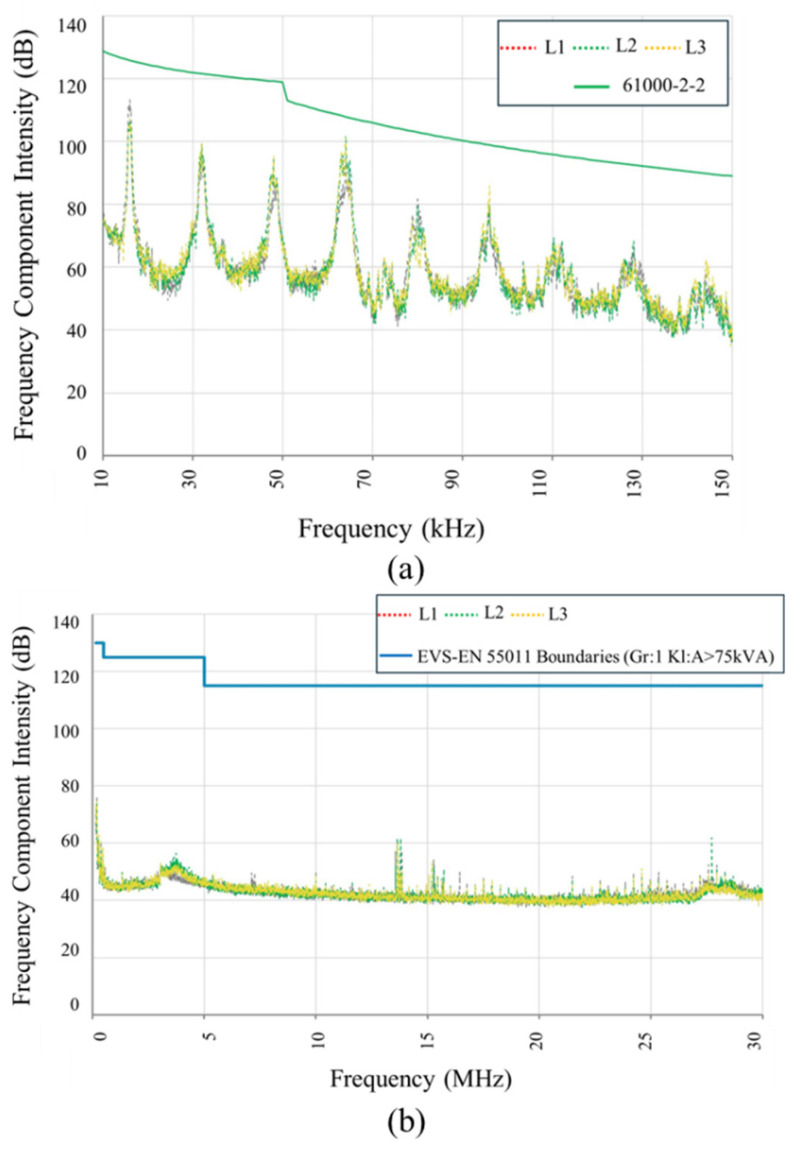
EMI measurements conducted during inverter operation at the Kopli Substation: (**a**) spectrum in the 9–150 kHz range, compared against IEC 61000-2-2 limits; (**b**) spectrum in the 150–30 MHz range, compared with EVS-EN 55011 Class A limits for >75 kVA equipment.

Extensive operational data were systematically collected, focusing on parameters critical to inverter integrity and compliance with power quality standards. Continuous measurements of three-phase voltage and current—both instantaneous and RMS values—enabled assessment of the inverter’s typical operating conditions, detection of transient events, and identification of fault-related signatures. A detailed harmonic analysis was conducted up to the 50th harmonic (2 kHz) to evaluate the presence of distortion-induced electrical stress. Phase voltage asymmetry was closely monitored to detect any imbalance conditions that could impose additional loading or instability on inverter operation. Furthermore, high-frequency conducted interference was assessed across the 9 kHz to 30 MHz spectrum using calibrated high-voltage probes, providing a precise characterization of electromagnetic disturbances potentially impacting inverter performance. It can be observed that the harmonics, both low frequency (150 kHz) and high frequency (30 MHz), are in the acceptable range, according to the boundaries defined by 6100-2-2 and EVS-EN55011 standards, as shown in Figure 8.

A targeted monitoring campaign was implemented to capture both nominal operating conditions and transient inverter fault events under realistic grid-connected scenarios. Central to this strategy was the use of a power quality analyzer equipped with advanced event-triggering algorithms, and configured to initiate high-resolution waveform recordings whenever instantaneous phase currents exceeded short-circuit thresholds (approximately >1500 A RMS). This enabled precise capture of critical transients associated with inverter damage. Data acquisition was conducted continuously for forty-five days, with regular maintenance and calibration ensuring data reliability and completeness. Special emphasis was placed on time windows surrounding inverter failures, enabling focused extraction and validation of fault-related data without disrupting plant operations. The recorded transients included detailed oscillograms, supporting post-event analysis of fault initiation, current dynamics, relay operation, and ground fault development. These signatures closely aligned with those obtained during laboratory fault simulations, providing a consistent diagnostic framework. This integrated monitoring methodology effectively bridged field data with experimental findings, enabling robust validation of the inverter failure mechanisms observed at the Kopli Solar Power Plant.

## 6. Field Results and Fault Signature Analysis

**Figure 9:**
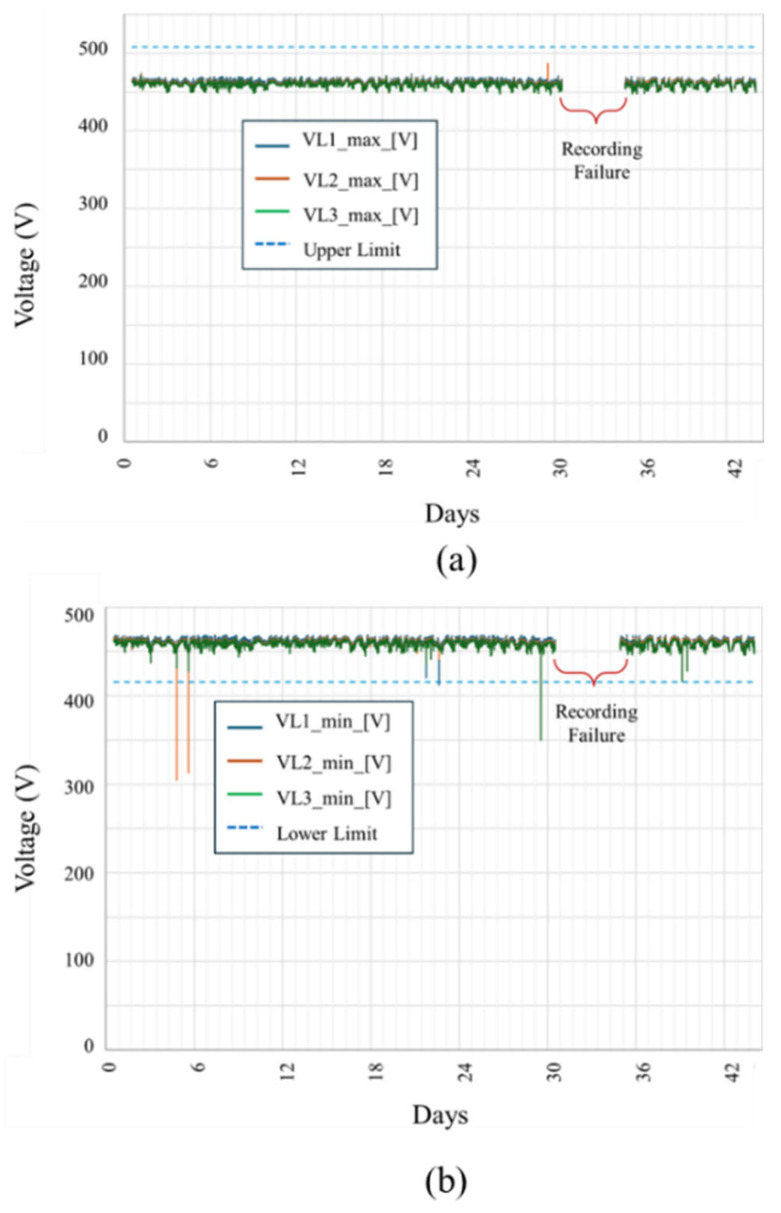
Recorded maximum and minimum phase voltages at the solar power plant during the monitoring period: (**a**) Peak phase voltages (10 ms max) for all three phases. No instances exceeded the upper limit of 508 V (+10% of the nominal 462 V). (**b**) Minimum phase voltages (10 ms max), showing several events below the lower limit of 416 V (−10% of nominal).

Continuous monitoring at the Kopli Solar Power Plant provided comprehensive operational data, enabling the accurate characterization of inverter performance during both regular and faulty conditions. Analysis of normal operation parameters showed voltages consistently within permissible ranges, typically within ±10% of the nominal 462 V line-to-neutral voltage. No instances were recorded in which the measured voltage exceeded the upper permissible limit (462 V + 10%). However, several instances were observed in which the voltage dropped below the lower permissible limit (462 V − 10%), as illustrated in Figure 9 and Table 1.

**Figure 10:**
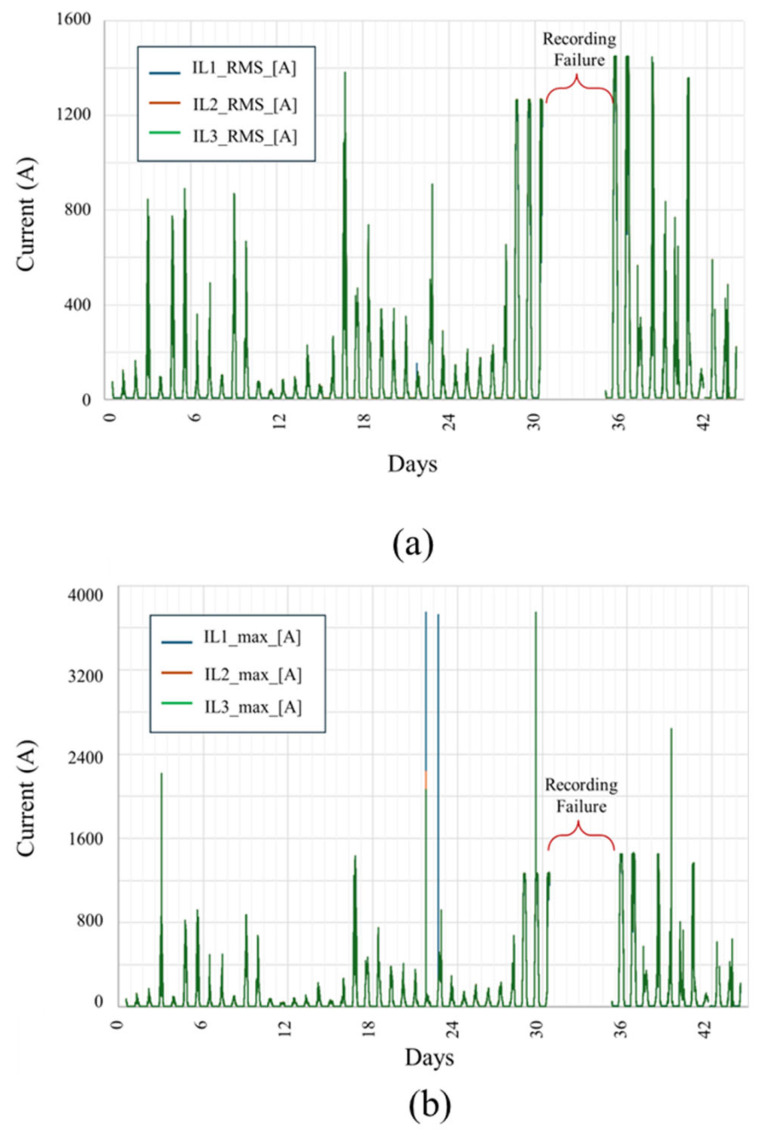
Current profiles recorded at the Kopli Solar Power Plant across three phases (IL1, IL2, and IL3) over a continuous monitoring period. (**a**) RMS operational current levels with values up to ~1500 A. (**b**) The instantaneous values capture severe overcurrent events exceeding 3500 A, corresponding to inverter fault conditions. Periods labeled “Recording Failure” indicate data loss due to equipment malfunction or transient disruptions in measurement continuity.

Figure 10 illustrates the RMS and peak current values recorded across three phases (IL1, IL2, and IL3) over a 44-day monitoring period at the Kopli Solar Power Plant.

Figure 10a shows the RMS values, within which current magnitudes generally remained below 1500 A, consistent with nominal inverter operation. Figure 10b presents the peak values (10 ms window), revealing multiple transient current spikes significantly exceeding 1500 A, with certain events surpassing 3000 A—indicative of short-circuit or fault conditions. These extreme peaks align with known failure events in the system. The exact time of current spikes is given in Table 2. A period of data recording failure is marked in both plots between days 30 and 36, where data was unavailable due to instrumentation issues.

**Figure 11:**
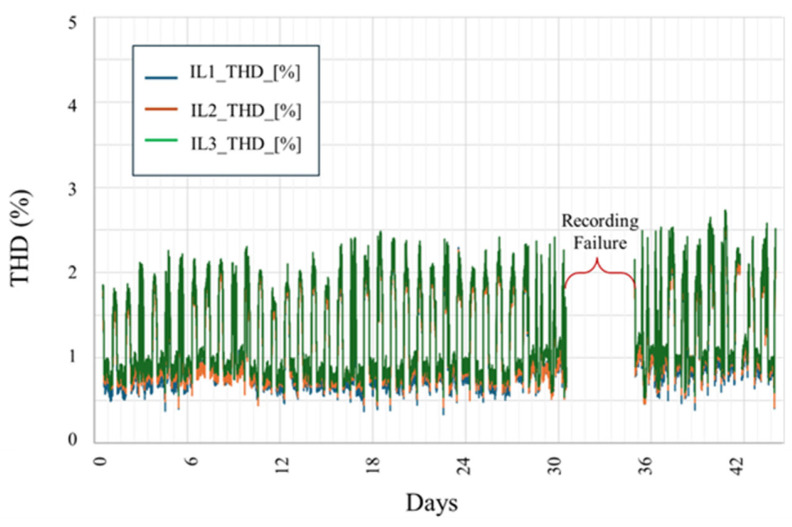
Three-phase total harmonic distortion (THD) profiles of line currents (IL1, IL2, and IL3) measured at the Kopli Solar Power Plant. The recorded THD values consistently remained below 3%, indicating satisfactory harmonic performance across the observation period.

Figure 11 illustrates the phase-wise Total Harmonic Distortion (THD) of the current waveforms (IL1, IL2, and IL3) recorded over the entire monitoring period. THD levels remained consistently low across all three phases, with IL1 and IL2 typically remaining below 1.5%, and IL3 exhibiting slightly higher distortion but generally staying under 4%. These values are well within the acceptable thresholds for industrial environments, indicating minimal harmonic distortion during normal operation.

**Figure 12:**
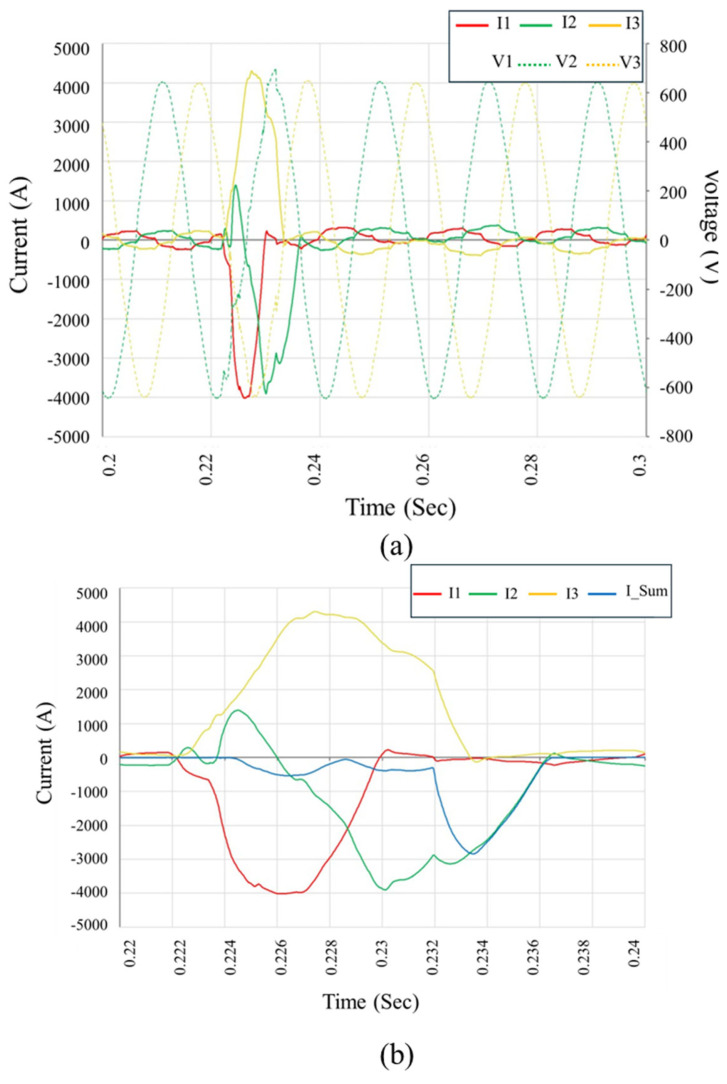
Field-recorded waveform during inverter failure event on 2 February 2024. (**a**) Phase voltages and currents showing a sudden three-phase short-circuit followed by rapid fault progression. (**b**) Detailed current analysis, revealing high-magnitude asymmetric fault currents and cumulative neutral current (I_Sum) indicative of ground fault and arc propagation. The sustained overcurrents confirm the delayed protection response and internal inverter damage.

High-resolution field monitoring identified transient fault waveforms that strongly correlated with catastrophic inverter failures. Figure 12 illustrates one such event, where a severe three-phase short-circuit initiates abruptly at approximately 0.223 s, and lasts for about 10 milliseconds, which corresponds to roughly 50% of a 50 Hz cycle. This short-duration but high-intensity event is characterized by a sharp, simultaneous rise in phase currents (I1, I2, and I3) and an immediate voltage collapse across all three lines (V1, V2, and V3), indicating a total loss of control within the inverter’s switching bridge. Figure 12a clearly shows the symmetric onset of the fault, followed by asymmetric propagation—particularly visible in the divergent decay rates of phase currents—implying the development of phase-to-ground conduction paths, likely due to internal arc formation and dielectric breakdown. Figure 12b provides a focused view of the individual phase currents and the composite neutral current (I_Sum). The persistent current imbalance and elevated I_Sum over several milliseconds indicate incomplete current cancellation, confirming the malfunction of internal protective elements such as isolation relays. The sustained arc energy observed during this interval suggests that the inverter’s internal disconnection logic failed to interrupt the fault swiftly, allowing further structural and thermal damage to propagate within the inverter enclosure.

**Figure 13:**
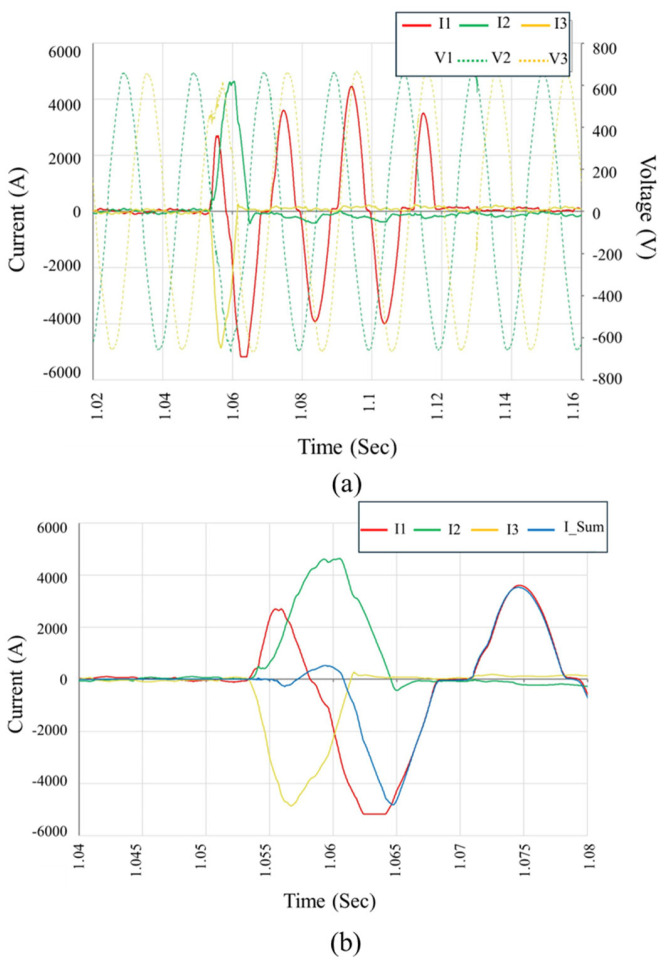
Waveform recordings from the inverter failure event on 24 February 2024 (Inverter #9). (**a**) Phase voltage and current waveforms showing the onset of a three-phase short-circuit followed by irregular arc behavior. (**b**) Zoomed-in current waveforms indicating sequential arc ignition and extinction across phases, with the cumulative neutral current (I_Sum) highlighting insulation breakdown and ineffective arc interruption. These patterns reflect repeated arc re-ignition consistent with internal insulation failure.

Figure 13 illustrates another fault event, captured during another instance of inverter failure. The waveform shows an abrupt current surge initiating around 1.056 s and sustained for approximately 10 ms, which corresponds to half of one 50 Hz cycle. During this short interval, the inverter output enters a high-energy fault condition characterized by near-simultaneous current escalation in all three phases, followed by distinct asymmetries in both magnitude and duration. Figure 13a depicts the phase currents (I1, I2, and I3) and corresponding voltages (V1, V2, and V3), in which the fault inception is marked by a collapse in line voltages and a steep rise in current, exceeding 6 kA in some phases. Such magnitudes strongly indicate the development of a full three-phase internal fault. In Figure 13b, the segregated current waveforms and their summation (I_Sum) provide further insights into the fault dynamics. An initial phase-to-phase short-circuit evolves into a phase-to-ground conduction path, as evidenced by the imbalance in I1 and I3 relative to I2 and the sustained deviation of I_Sum from zero. This confirms that the internal protection logic once again failed to clear the fault rapidly, permitting high arc energy to propagate and cause irreversible component damage. These waveforms reinforce the hypothesis that an internal switching device failure, possibly triggered by an upstream transient or design vulnerability, resulted in simultaneous semiconductor conduction and uncontrolled current looping within the inverter bridge.

**Figure 14:**
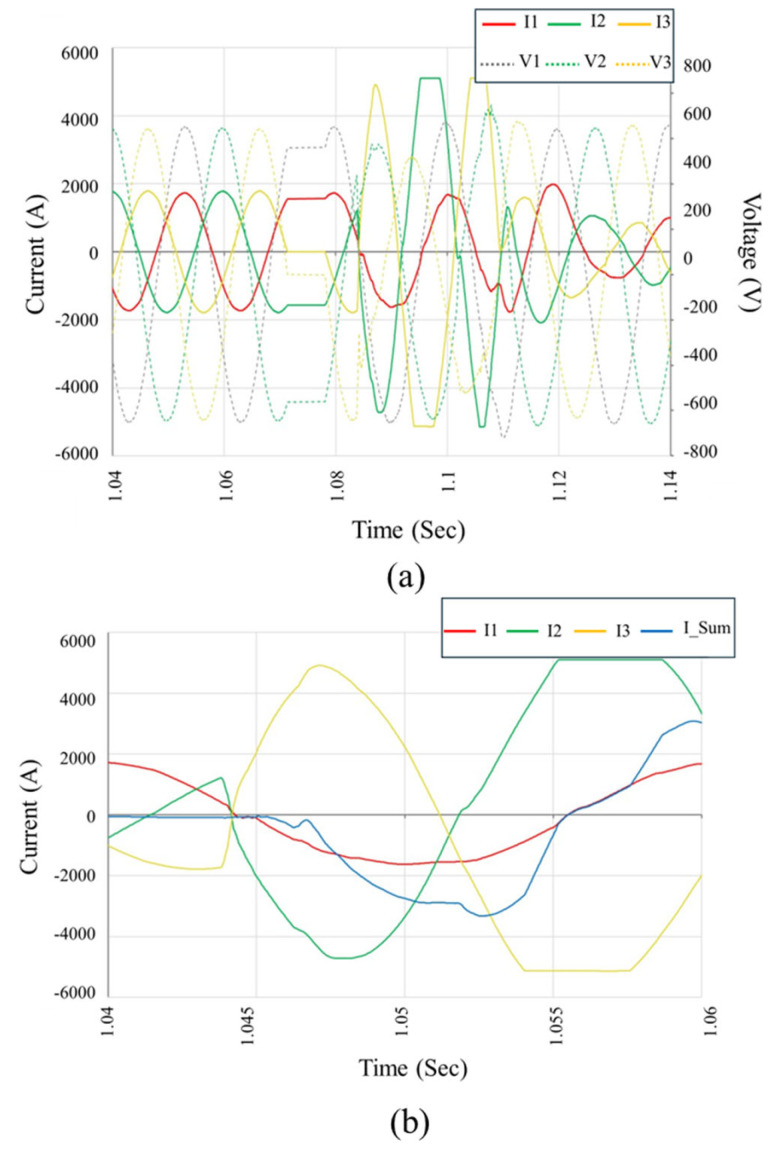
Recorded waveforms from the inverter fault event on 4 March 2024. (**a**) Phase voltage and current waveforms indicating a two-phase fault transitioning into an earth fault, with one phase remaining initially unaffected. (**b**) Current waveform detail showing unbalanced high-magnitude fault currents and rising neutral current (I_Sum), suggesting localized insulation failure and delayed isolation response within the inverter.

Figure 14 presents another representative fault waveform captured during inverter operation. The fault onset occurs at approximately 1.048 s and persists until around 1.056 s, spanning a duration of roughly 8 ms, which corresponds to about 40% of a 50 Hz power cycle. Figure 14a displays a concurrent surge in all three phase currents (I1, I2, and I3), reaching peak values close to 6 kA and accompanied by a noticeable distortion and partial collapses in line voltages (V1, V2, and V3). Initially, the current escalation appears asymmetrical, particularly with I2 and I3 exhibiting abrupt growth, suggesting a developing phase-to-phase short. As the event progresses, the voltage waveform undergoes further deformation, and the current paths diverge in both amplitude and timing, which is indicative of an evolving internal arc propagation across phases. Figure 14b, which details the individual phase currents and the resulting neutral current (I_Sum), reinforces the interpretation of a severe fault. A pronounced deviation of I_Sum from zero throughout the fault window indicates a significant current imbalance and confirms the formation of an ungrounded loop or phase-to-ground path. Notably, this event lacks rapid attenuation, reinforcing the hypothesis of relay non-operation or delayed isolation due to failure of the internal protection mechanisms. The combination of high-magnitude current peaks, asymmetric current flow, and persistent I_Sum strongly suggests that this event has the same root cause as the previously observed failures: internal breakdown in the inverters’ power switching stage, likely accelerated by insufficient arc suppression and improper Fault Ride Through logic design.

Taken together, Figure 12, Figure 13 and Figure 14 validate the laboratory-identified failure modes under field conditions. They illustrate how negative terminal DC-side faults can trigger internal overcurrent conditions that evolve into multi-phase arc faults, leading to widespread damage due to the inverter’s delayed or inadequate protective response.

A critical analysis of the recorded waveforms and fault signatures uncovered significant deficiencies in inverter internal protective responses. In all documented short-circuit events, particularly those involving severe three-phase and subsequent ground faults, inverter protection mechanisms consistently demonstrated inadequate responsiveness. Specifically,

- Protective isolation relays, although activated, failed to effectively interrupt high-energy short-circuit currents rapidly enough, enabling prolonged electric arc formation and substantial internal component damage. Visual inspections corroborated relay malfunctions, clearly evidenced by severe relay thermal damage, indicating insufficient relay sizing or inadequate operational speed relative to internal fault energy release.
- Semiconductor switching components (IGBTs) consistently demonstrated catastrophic failure patterns, as documented in the waveform data, which suggested uncontrolled semiconductor conduction following internal faults. The observed waveforms showed sustained current conduction even after fault initiation, strongly suggesting inadequate semiconductor protection, delayed internal protective logic activation, or ineffective fault-detection circuits.
- Arc dynamics revealed by oscillograms further demonstrated protective inadequacies. Sustained arc conditions were evident across multiple events, clearly indicated by repeated high-current pulses and subsequent arc re-ignition occurrences. These repetitive arc events strongly indicated compromised internal insulation integrity and ineffective or absent arc-fault interruption mechanisms within the inverter protective circuitry.

In summary, the recorded waveform data from critical transient events clearly demonstrated that the Sungrow SG125HX inverters at Kopli lacked sufficiently robust internal protective responses. Slow protective relay response times, semiconductor device vulnerabilities, and inadequate arc mitigation strategies were conclusively identified as key factors exacerbating the internal damage, which ultimately resulted in inverter destruction. These field findings provided empirical validation of laboratory simulations, significantly clarifying failure propagation mechanisms and highlighting critical design areas requiring urgent attention and improvement.

## 7. Integrated Discussion and Comparative Validation

This systematic investigation, encompassing both laboratory simulations and detailed field monitoring at the Kopli Solar Power Plant, provided convergent evidence clearly confirming the root cause mechanisms behind repeated, catastrophic inverter failures. Laboratory-based fault simulations established that negative DC terminal short-circuits consistently triggered severe, uncontrolled internal fault currents exceeding 1 kA instantaneous magnitude. Critically, laboratory testing revealed that protective systems within the inverter demonstrated inadequate responsiveness, allowing fault conditions to persist long enough to cause irreversible damage to internal semiconductor components and isolation relays.

The field observations remarkably mirrored these laboratory results, as evidenced by the recorded transient events. The field oscillograms consistently showed instantaneous multi-phase faults initiated simultaneously, rapidly transitioning into catastrophic ground faults marked by repeated, high-intensity arc events. These fault patterns closely replicated the laboratory-observed behavior under negative DC-side short-circuit conditions. Moreover, extensive component damage documented in visual inspections corroborated waveform analyses, strongly suggesting that negative DC input faults served as the primary triggering event for severe multi-phase short-circuits and subsequent grounding arcs.

This comprehensive comparative assessment conclusively indicates a fundamental design vulnerability, one specifically concerning the inverter’s internal response to DC-side faults, rather than external grid or environmental disturbances alone.

Through rigorous cross-validation, the integrated analysis confirmed two primary interrelated root causes underlying the inverter destructions observed:

- Negative DC Input Short-Circuit Fault as Primary Trigger: Laboratory experiments explicitly identified that grounding faults on the negative DC input terminal consistently caused immediate and catastrophic inverter internal faults. This observation was subsequently validated by field data, as evidenced by consistent patterns of rapid multi-phase current escalation and arc propagation, strongly indicative of negative DC-side induced faults.
- Delayed or Inadequate Inverter Internal Protection Response: The waveform data from both the laboratory and the field environments explicitly demonstrated delayed protective response times specifically involving isolation relays and semiconductor switching components. The protective circuitry repeatedly failed to disconnect the faulty circuits quickly enough to prevent high-energy arc events and extensive thermal destruction, thus exacerbating the internal damage significantly.

The broader implications of these findings for inverter design standards and operational best practices are described in the following.

These findings have critical implications, both at the inverter component level and for broader industry design and operational guidelines. Firstly, the demonstrated inadequacy of the protective responses highlights an urgent need to revisit inverter internal protection standards, specifically, those targeting DC-side faults. While current compliance standards (e.g., the IEC 62109 and IEC 61000 series) address general inverter safety and electromagnetic compatibility, these standards currently lack detailed and stringent criteria explicitly addressing inverter behavior under extreme DC input faults. Therefore, current certification and testing procedures should incorporate specific DC-side fault-response validation, particularly targeting rapid internal short-circuit detection, effective arc mitigation, and immediate disconnection protocols.

Operationally, these findings underscore the necessity of enhancing monitoring strategies in utility-scale PV installations, focusing explicitly on DC-side operational parameters. Such strategies should involve advanced fault detection and early-warning systems, integrated within inverter control firmware and capable of rapidly isolating faults and preventing catastrophic escalation. Additionally, industry-wide adoption of improved maintenance practices and regular DC-side insulation integrity tests can significantly reduce the probabilities of occurrence and potential damage severity levels associated with similar inverter faults.

Based on the comprehensive insights derived from laboratory simulations and validated through field analysis, several essential inverter design modifications and protective strategy enhancements are proposed for adoption by inverter manufacturers and solar plant operators:

- Enhanced DC Input Protection Mechanisms: Implementation of rapid-response isolation relays specifically engineered for DC-side faults, including dedicated semiconductor fuse protections and high-speed circuit interruption mechanisms intended to minimize internal arc durations and energy release.
- Advanced Fault Detection and Control Algorithms: Integration of intelligent inverter control algorithms utilizing real-time DC-side current and voltage feedback, ensuring immediate detection and isolation of short-circuit conditions. These algorithms should incorporate adaptive fault-response logic and be capable of instantly triggering inverter shutdowns or selective internal component isolations under fault conditions.
- Improved Arc-Fault Mitigation Design: Reinforcement of inverter internal insulation systems, using advanced insulation materials or coatings to reduce susceptibility to moisture ingress and insulation breakdown. Additionally, the integration of arc-resistant materials and physical barriers within critical inverter compartments can substantially reduce damage propagation when arcs occur.
- Enhanced Relay and Semiconductor Component Specifications: Revision of inverter internal component specifications, ensuring robust design margins that can safely withstand transient high-current conditions associated with DC-side faults. Specifically, the incorporation of semiconductor switching devices and relays rated for substantially higher instantaneous currents, coupled with optimized thermal management systems, could significantly enhance inverter resilience.

Collectively, these recommended modifications represent a systematic response to the inverter vulnerabilities identified, providing practical, industry-wide pathways toward significantly improved inverter reliability, safer operational conditions, and sustained performance under challenging DC-side fault scenarios.

## 8. Conclusions

This comprehensive investigation systematically examined recurrent catastrophic inverter failures at the Kopli Solar Power Plant (PEJ), Estonia, utilizing integrated field-based monitoring and controlled laboratory testing. Multiple hypotheses were evaluated through rigorous analysis, and findings were supported by extensive field measurements and laboratory validation. Field measurements revealed that general operational parameters, such as grid voltage levels, voltage harmonic distortion, and phase imbalance remained consistently within permissible operational limits. Notably, the voltage harmonic distortion and phase asymmetry recorded were well below the industrial standard limits, confirming that routine external grid conditions, including moderate voltage fluctuations, did not trigger inverter failures directly. However, the detailed waveform analyses field-recorded during fault events consistently showed abrupt, severe multi-phase short-circuits accompanied by sustained arcs, indicative of internal inverter faults rather than external grid anomalies. These waveform signatures closely matched conditions replicated during the controlled laboratory testing, conclusively demonstrating that inverter faults originated internally, and were specifically triggered by negative DC input-to-ground short-circuits.

Laboratory measurements further confirmed that negative DC input grounding consistently resulted in catastrophic faults characterized by high current magnitudes exceeding semiconductor tolerances, prolonged internal arcs, and eventual component destruction. Contrarily, laboratory tests revealed that positive DC input grounding scenarios activated effective inverter protection functions, underscoring the vulnerability specifically associated with negative DC grounding events. Critical weaknesses in inverter internal protective mechanisms were identified. These included delayed or ineffective semiconductor switching responses, inadequate relay interruption capabilities, and insufficient arc-suppression strategies. These failures of internal protection led to extended fault durations, resulting in severe thermal damage and permanent inverter component degradation.

Based on these clearly validated results, the following targeted recommendations have been formulated.

For Inverter Manufacturers:

- Implement reinforced, internal DC fault-detection systems designed specifically to mitigate negative DC-side faults.
- Enhance relay response speed and reliability and select semiconductor switching components with higher transient fault tolerance.
- Adopt a more robust insulation and moisture-ingress protection to prevent internal arc initiation and propagation.

For PV System Designers:

- Integrate proactive inverter protection measures at the system design stage, emphasizing continuous DC-side parameter monitoring.
- Specify inverters with superior insulation standards and advanced internal protection features, particularly for utility-scale PV plants prone to DC-side grounding faults.

For PV Plant Operators:

- Conduct periodic detailed inspections and testing of inverter internal protective functions, focusing on DC-side insulation integrity.
- Employ predictive and condition-based monitoring systems capable of detecting early signs of insulation breakdown or protective component degradation, thus mitigating catastrophic inverter failures.

In conclusion, this research provides critical insights into the internal failure mechanisms of photovoltaic inverters, demonstrating through laboratory and field evidence that internal DC-side insulation faults, rather than external grid disturbances, represent the primary catalyst for the catastrophic inverter failures at Kopli PEJ. Addressing these inverter-internal vulnerabilities through improved design, comprehensive protection strategies, and enhanced operational practices is essential to significantly enhance inverter reliability, system stability, and the long-term economic sustainability of photovoltaic installations.

## Figures and Tables

**Table 1:** Times and specifications of recorded phase voltage dips.

Time	Registered Voltage	Comment
04.02.2024 12:48	305 V	Voltage sag originating in the transmission system.
05.02.2024 13:22	313 V	Voltage sag originating in the transmission system.
25.02.2024 09:15	413 V	Short-circuit condition, also registered with short-circuit current condition.
04.03.2024 11:00	350 V	Short-circuit condition, also registered with short-circuit current condition.
15.03.2024 15:47	417 V	Short-circuit condition, also registered with short-circuit current condition.

**Table 2:** Times and specifications of recorded current spikes.

Time	Registered Current	Comments
02.02.2024 12:48	2.2 kA	Transient Short-Circuit
24.02.2024 09:39	3.8 kA	Primary Failure
25.02.2024 09:25	Over 3.7 kA	Transient Short-Circuit
04.03.2024 11:00	Over 3.7 kA	Insulation Failure
15.03.2024 15:47	2.6 kA	Transient Short-Circuit

## Data Availability

Restrictions apply to the datasets.
